# Examining the Impact of Supramaximal Eccentric and Long‐Lever Isometric Training Protocols on Isometric Hamstring Strength, Nordic Hamstring Performance, and Sprint Speed

**DOI:** 10.1002/ejsc.70257

**Published:** 2026-09-29

**Authors:** Adam E. Sundh, Devin S. VerVoort, Nicholas J. Ripley, A. J. Lamb, Paul Comfort

**Affiliations:** ^1^ School of Health and Society University of Salford Salford UK; ^2^ Chicago Bears Football Club Lake Forest Illinois USA; ^3^ Department of Athletics Purdue University Northwest Hammond Indiana USA; ^4^ School of Medical and Health Sciences Edith Cowan University Joondalup Australia

**Keywords:** eccentric hamstring strength, isometric hamstring strength, nordic hamstring exercise, posterior chain training, sprint performance

## Abstract

The aim of this study was to compare the effects of a 6‐week higher‐volume eccentric training protocol with a combined lower‐volume eccentric and long‐lever isometric protocol on posterior chain strength and sprint performance. Thirty‐three NCAA Division II soccer players (men: *n* = 17, age = 19.7 ± 1.2 years, height = 178.9 ± 6.6 cm, body mass 79.8 ± 8.1 kg; women: *n* = 16, age = 20.1 ± 0.9 years, height = 166.7 ± 5.2 cm, body mass = 67.0 ± 12.1 kg) were allocated to either group via exhaustive minimization. Nordic hamstring exercise break‐point angle (NBPA), rapid isometric hamstring strength (LLB), 20m sprint performance, and continuous angular knee extension velocity waveforms were assessed pre‐ and post‐intervention, and following a 2‐week detraining period. A significant main effect of time was observed for sprint, NBPA, and rapid isometric strength (*p* < 0.05); however, Bonferroni‐corrected within‐group comparisons reached significance only for sprint performance in both groups, with no significant between‐group differences for any outcome. Equivalence testing supported comparable sprint adaptations between protocols but could not be established for eccentric or isometric hamstring strength. All discrete outcome measures were largely maintained or continued to develop following the detraining period though range‐specific eccentric strength appeared more sensitive to the training cessation. A combined low‐volume eccentric and isometric training approach may represent a viable alternative to higher‐volume eccentric programming for hamstring strength and speed development, although this should be approached cautiously to preserve neuromuscular qualities relevant to hamstring injury risk mitigation.

## Highlights

- A low‐volume combined eccentric‐isometric hamstring strength protocol produced sprint adaptations comparable to a higher‐volume eccentric‐only protocol
- Eccentric and isometric hamstring strength adaptations were largely similar between groups, supporting flexibility in exercise selection for strength development
- Terminal‐range eccentric strength improved only in the high‐volume NHE group but reverted after detraining, suggesting this quality may require a minimum eccentric dose to be maintained

## 1 Introduction

Despite decades of research into hamstring strain injury (HSI) mitigation, the precise mechanical nature of hamstring function during maximal sprinting, and therefore the ideal training stimuli to mitigate injury risk, remains contested (Kalkhoven et al. 2023; Van Hooren and Bosch 2017). However, it is well established that HSIs represent one of the most prevalent and recurrent injuries across field‐based sports (Ekstrand et al. 2022; Maniar et al. 2023), driving considerable research into training interventions capable of reducing their occurrence (Lauersen et al. 2014; van Dyk et al. 2019). Eccentric strength training, particularly through the Nordic hamstring exercise (NHE), has emerged as one of the most extensively evidenced approaches to HSI mitigation, with combined intervention protocols such as the FIFA 11+ warm‐up program similarly demonstrating reductions in HSI incidence (van Dyk et al. 2019). However, when analysis is restricted to randomized control trials only, the evidence underpinning the protective effect of the NHE remains inconclusive and at best conditional (Impellizzeri et al. 2021). Nonetheless, the available evidence does appear to favor the NHE over no intervention or alternative modalities, and the NHE has consistently demonstrated improvements in both eccentric hamstring strength (Medeiros et al. 2020), and biceps femoris long head (BF_LH_) fascicle length (Andrews, Pai, et al. 2025), two of the primary modifiable risk factors associated with HSI occurrence (Andrews, Shield, et al. 2025; Timmins et al. 2016). Importantly, Cuthbert et al. (2020) demonstrated that reducing NHE volume prescription does not negatively impact adaptations in eccentric strength or muscle architecture when compared to higher‐volume equivalents, with weekly volumes across the literature ranging from 21 to 73 repetitions per week, and suggesting that lower‐volume approaches may be more appropriate for athletes with the potential to improve intervention compliance (Ripley et al. 2021), and perhaps allow for the introduction of additional or complementary exercise stimuli to further enhance adaptations.

A growing body of evidence has highlighted the potential benefits of training at longer muscle lengths (Wolf et al. 2023, 2025), with Maeo et al. (2024) demonstrating that lengthened‐state training (accentuated hip‐flexed position) induced greater increases in overall hamstring strength and BF_LH_ muscle volume compared to the NHE, alongside greater increases in aponeurosis area. This may be particularly relevant given that the BF_LH_ appears to be the most commonly injured hamstring musculature (Grange et al. 2023). Training at longer muscle lengths may therefore represent an additional adaptive stimulus for both HSI mitigation and sprint performance, as architectural properties of the hamstrings are associated with sprinting biomechanics (Ripley et al. 2025). Maximal isometric contractions (i.e., overcoming or pushing isometrics) may also represent a viable complementary training stimulus, given the maximal voluntary effort required by both modalities. In this regard, an isometric long‐lever bridge, performed at long muscle lengths, has demonstrated maximal BF_LH_ activation at 30 degrees of knee flexion (Hirose and Tsuruike 2018), suggesting it may be a particularly effective exercise for targeting the most vulnerable hamstring musculature. Furthermore, given that isometric training is typically associated with much less delayed onset muscle soreness compared to eccentric training (Philippou et al. 2004), a common barrier to implementation of the NHE in sporting environments (Bahr et al. 2015), it may represent a particularly well‐suited additive stimulus with the potential to improve both strength characteristics whilst maintaining intervention tolerability and compliance. Therefore, the aim of this study was to compare a higher‐volume eccentric NHE protocol to a lower‐volume NHE protocol combined with a maximal isometric protocol to determine whether a modified, potentially more tolerable prescription could produce comparable or superior adaptations to a more traditional, higher‐volume eccentric approach. It was hypothesized that both protocols would produce significant improvements across all outcome measures, but that the combined group would demonstrate non‐inferior adaptations despite the lower eccentric volume.

## 2 Materials and Methods

### 2.1 Study Design

A quasi‐experimental design, utilizing exhaustive allocation minimization for group assignment and a pre‐test, post‐test, and de‐training follow‐up structure, was employed to compare the effects of a higher‐volume eccentric NHE protocol to a lower‐volume NHE protocol combined with a maximal isometric protocol on hamstring strength and sprint performance. Following baseline testing, in which participants completed multiple repetitions of each measure, including the 20 m sprint, the isometric single‐leg long‐lever bridge (SL‐LLB), and the NHE, participants were allocated to one of two training groups via exhaustive allocation minimization. This iterative optimization approach evaluates every possible grouping combination to identify the allocation that simultaneously minimizes between‐group differences across both mean and peak values of each baseline measure, ensuring the most balanced possible group allocation at baseline and reducing the likelihood that observed post‐intervention differences could be attributed to pre‐existing between‐group disparities. Participants then completed a 6‐week training intervention based on their allocated group, following which post‐intervention testing was conducted using identical protocols to baseline. A 2‐week de‐training period subsequently followed, after which a final re‐test was performed to determine whether training‐induced adaptations were retained or reversed following the cessation of training. A visual overview of the study design is presented in Figure 1.

**FIGURE 1:**
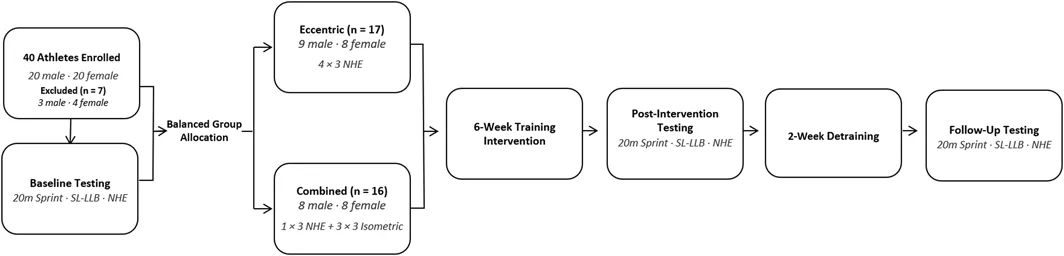
Timeline of the study. Illustration of the training and assessment periods across the two groups.

### 2.2 Participants

Forty NCAA Division II soccer players (20 men, 20 women) volunteered to participate in this study; however, following dropouts due to illness, injury, personal reasons, or scheduling conflicts, 33 players completed the study consisting of men (*n* = 17, age = 19.7 ± 1.2 years, height = 178.9 ± 6.6 cm, body mass 79.8 ± 8.1 kg) and women (*n* = 16, age = 20.1 ± 0.9 years, height = 166.7 ± 5.2 cm, body mass = 67.0 ± 12.1 kg). All athletes were identified as tier 3 according to the classification tier system suggested by McKay et al. (2022). Following the allocation minimization procedure described above, participants were separated into two balanced groups (combined protocol and eccentric protocol), consisting of 16 and 17 athletes, respectively. Group‐specific descriptive characteristics are provided in Table 1.

**TABLE 1:** Baseline participant characteristics and between‐group comparisons.

Variable	Combined protocol (mean ± SD)	Eccentric protocol (mean ± SD)	*p*	Hedges' *g*
Age (years)	19.94 ± 0.94	19.84 ± 1.26	0.8	0.09
Height (cm)	174.13 ± 7.90	171.25 ± 9.01	0.3	0.33
Weight (kg)	73.25 ± 10.24	73.25 ± 13.85	1	0.01

*Note: p*‐values from independent samples *t*‐test; Hedges' *g* indicates effect size. Groups did not differ significantly on any characteristic (all *p* > 0.05).

Participants were required to have had no posterior‐chain‐related injuries within the preceding 6 months before taking part in the study. Organizational consent was acquired prior to approaching the participants, and all participants provided written informed consent to participate in the study. Ethical approval was granted by the University of Salford's institutional ethics committee in accordance with the Declaration of Helsinki (reference no: #2025‐7488‐9326, approval date: August 5th, 2025). An a priori sample size calculation was conducted to determine adequate power for paired group contrasts using expected effect sizes derived from previous work employing similar eccentric or isometric training interventions of the posterior chain musculature (Carmichael et al. 2022; Presland et al. 2020, 2018). The calculation assumed a Cohen's *d* of 0.60, statistical power of 0.80, and an alpha level of 0.05, resulting in a minimum sample size of 11 participants per group.

### 2.3 20‐m Sprint

Linear sprint performance was assessed over a distance of 20 m using electronic timing gates (Brower Timing Systems, Draper, UT, USA) set at a height of 1.0 m. Participants began each trial from a standing start, positioned 0.30 m behind the start gate to prevent premature trigger activation, with a standardized two‐point staggered stance adopted for all trials. Prior to testing, a standardized warm‐up was completed, comprising three progressive sprints performed at approximately 50%, 75%, and 90% of maximal effort. Each participant then completed two maximal sprint trials, with 2 minutes of passive recovery permitted between efforts.

### 2.4 Isometric Long‐Lever Bridge

The SL‐LLB was assessed using Hawkin Dynamics force plates (Hawkin Dynamics Inc., Portland, ME, USA) sampling at 1000 Hz, with data collected via the Hawkin Dynamics mobile application. Participants were positioned supine with their heels in direct contact with the force plates, as described previously (Sundh et al. 2026a, 2026b). The inferior angle of the scapula was supported on a 15.24 cm (6‐inch) foam platform to elevate the torso and facilitate the hip extension intent during the assessment. A loaded barbell, with a rigid foam pad positioned at the inguinal fold, was used to anchor the lower extremity and provide a point of resistance that participants drove against. A knee angle of 30° and a hip angle of 135 ± 5° were standardized across all trials using a goniometer prior to each repetition. Prior to each trial, slack in the barbell was removed to establish a standardized pre‐tension against the loaded barbell, ensuring a consistent starting point for force application across repetitions. Upon visual cueing, signaled by a brief white flash, participants were directed to push as hard and as fast as possible by driving their heels into the force plates and extending their hips into the barbell, maintaining maximal effort for 3–5 seconds. Strong verbal encouragement was provided throughout each trial, and a rest period of 30 seconds was given between trials. Hip and knee joint angles were re‐measured between trials to ensure consistent positioning was maintained. Rapid force production was reported from the force‐time curve, defined as the force produced at 150 ms from the onset of force production (determined as the point at which force exceeded ± 3 standard deviations (SD) of the average force recorded during the one‐second baseline weighing phase (Guppy et al. 2024)), representing the earliest reliable force‐time point for this assessment (Sundh et al. 2025).

### 2.5 Nordic Break‐Point Angle

The Nordic hamstring break‐point angle, derived from the NHE, was assessed using an iPhone camera filmed at 4K resolution and 60 frames per second (sufficient for a low velocity task) positioned in the sagittal plane at a distance of 5 m from the participant. Each participant performed three trials of the NHE. Participants assumed a kneeling position with their heels secured beneath a padded attachment fixed to a squat rack, which served as the anchoring resistance. Participants were instructed to maintain their hips fixed in line with the knee and shoulder joints throughout the movement. From this position, participants performed a forward‐falling action, lowering the head, trunk, and thighs via knee extension until they could no longer resist the gravitational load, with their hamstrings providing the primary resistance. Kinematic analysis was subsequently performed using Kinovea software (version 2025.1.1). Although a velocity threshold of 10°·s^−1^ was among the earliest criteria to operationalize the break‐point (Lee et al. 2018, 2017), this criterion was not deemed appropriate for the current cohort, as angular knee extension velocities at or near this threshold were observed throughout the majority of the sequence, rather than at a discrete transition point. Moreover, given that prior research has identified 30°·s^−1^ as indicative of a controlled eccentric phase (Nishida et al. 2022), corresponding to approximately 3 seconds of lowering, a threshold of 10°·s^−1^ would imply a NHE of roughly 9 seconds in duration, which would not be considered representative of typical performance. A threshold of 30°·s^−1^ has therefore also been adopted in subsequent work (Soga et al. 2023), with even more recent work adopting a threshold of 24.7°·s^−1^ (Soga et al. 2026) by the same authors, indicating that the appropriate threshold may be cohort‐specific rather than fixed across populations. Therefore, the break‐point was instead operationalized as the joint angle at the point of inflection identified via a velocity‐to‐joint angle ratio of the kinematic time series, whereby the first derivative of this ratio was used to locate the instant at which angular knee extension velocity began to rise disproportionately relative to the knee joint angle, consistent with the onset of voluntary muscular failure of the hamstrings (Figure 2).

**FIGURE 2:**
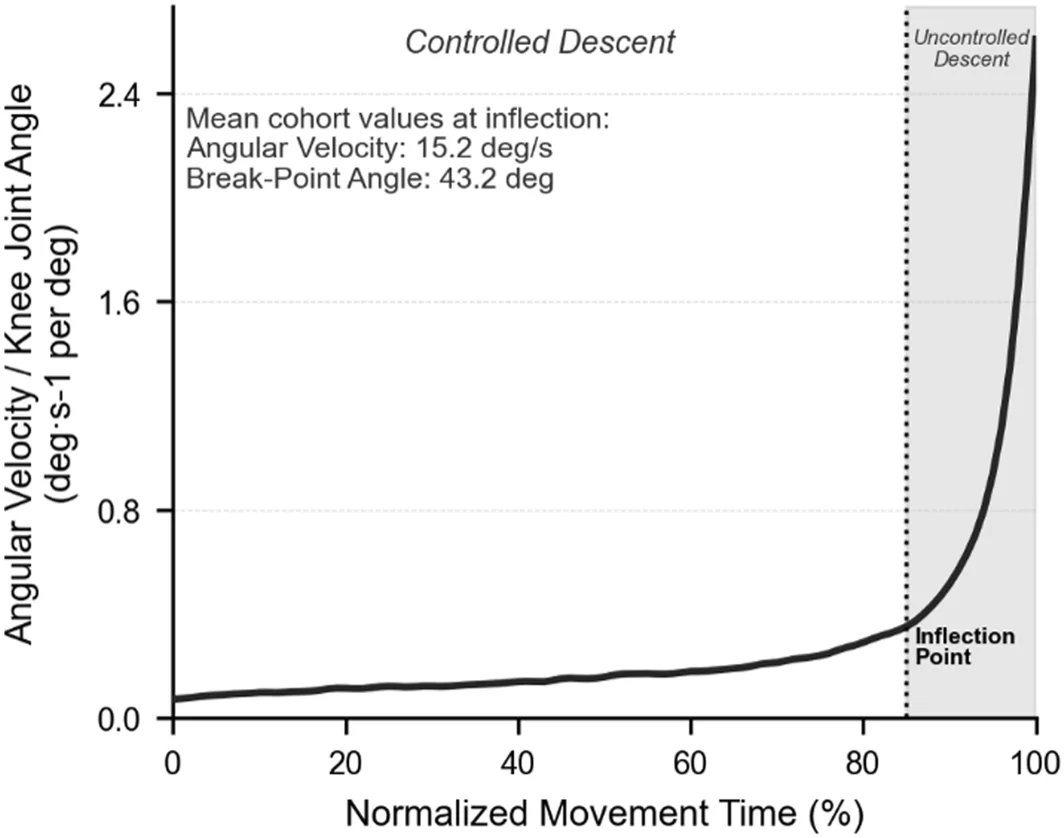
Identification of the Nordic break‐point angle. Velocity‐to‐angle ratio across the normalized NHE, collapsed across all participants, groups, and time points. The dotted vertical line denotes the inflection point, identified as the moment at which the velocity‐to‐angle ratio transitions from a period of gradual increase to rapid acceleration, delineating the boundary between the controlled descent phase and the uncontrolled portion of the movement.

### 2.6 Reliability

Within‐session reliability of the primary outcome measures (ICC(3,1) and CV%), calculated from repeated baseline trials, is reported in Table S1, with all outcomes meeting acceptable reliability thresholds.

### 2.7 Nordic Hamstring Kinematic Analysis

To characterize the kinematic profile of the NHE across training conditions and time points, a continuous time series of angular knee extension velocities was extracted for each trial and time‐normalized to 0%–100% of the movement duration. Previous evidence indicates that the NHE only selectively loads the BF_LH_, the hamstring muscle most implicated in HSI (De Smet and Best 2000), only as the knee approaches terminal extension (Hirose et al. 2021; Soga et al. 2021; Suskens et al. 2023; Van Hooren et al. 2022), such that participants unable to resist into this range may predominantly stress the semitendinosus and semimembranosus, respectively, limiting the exercise's specificity. Although the break‐point angle partially addresses this by indexing the degree of knee extension achieved before voluntary muscular failure, and therefore whether participants entered the range of presumed BF_LH_ loading, it does not capture how the velocity of descent was modulated across the full movement, nor whether adaptations were confined to the terminal phase or distributed more broadly across the repetition. Continuous analyses of the angular velocity waveforms were therefore employed to examine whether training altered the velocity of descent across the full repetition, and specifically to verify whether the rate of descent through the terminal phase was reduced following the intervention, reflecting a greater capacity to sustain eccentric forces in the terminal range.

### 2.8 Eccentric Intervention

Participants assigned to the higher‐volume eccentric protocol continued their existing strength and conditioning programming throughout the intervention period without modification, with two exceptions: no additional posterior chain exercises were performed outside of the prescribed NHE sessions, and participants refrained from any structured sprint training for the duration of the intervention. This restriction was implemented to isolate the training stimulus and to avoid confounding adaptations with those attributable to the protocol. In addition to their regular programming, participants performed supervised NHE sessions twice per week for 6 weeks, with a minimum of 48 hours separating each session. Each session consisted of four sets of three repetitions of the NHE, performed at maximal eccentric intensity, with participants instructed to resist as long as possible during the descent. Despite representing the higher‐volume condition within the present study (24 weekly repetitions), this prescription remains relatively low compared to the volume identified in the broader literature, typically ranging between 21 and 73 total weekly repetitions (Cuthbert et al. 2020). Nonetheless, to ensure progressive overload across the intervention, if a participant completed any set without reaching voluntary muscular failure, that is, demonstrated adequate strength to maintain full control during the last 10°–20° of knee extension during the NHE (Bourne et al. 2017; Duhig et al. 2019; Ripley et al. 2023), 5 lbs (2.26 kg) of additional external load was added in incremental stages until the participant could no longer complete the repetitions without technique breakdown. This approach ensured that each training session represented a maximal eccentric stimulus relative to each participant's capacity.

### 2.9 Combined Intervention

Participants assigned to the combined protocol similarly continued their existing strength and conditioning programming without modification and were subjected to the same restrictions as the eccentric group; no additional posterior chain exercises were performed, and structured sprint training was suspended for the duration of the intervention. This group performed the same twice‐weekly supervised sessions for 6 weeks with a minimum of 48 hours between sessions; however, the eccentric volume was reduced to one set of three repetitions of the NHE, performed under the same maximal eccentric conditions and criteria described above for the eccentric group. Following the eccentric component, participants performed an overcoming isometric in the long‐lever bridge position as described above. Unlike the assessment protocol, joint angles were not standardized via goniometry during training sessions. Instead, participants were instructed to maintain a minimally bent knee position, approximating a long‐lever configuration, reflecting the practical reality that precise joint angle measurement is not feasible in applied environments where multiple athletes perform the exercise concurrently. All participants performed three sets of three repetitions per leg, with each repetition sustained for 3 seconds, and were instructed to initiate each repetition as rapidly and forcefully as possible.

### 2.10 Statistical Analyses

Statistical analyses were performed using Python 3.12.7 (Python Software Foundation, DE, USA). All data were examined for normality prior to analysis using the Shapiro‐Wilk test. Descriptive statistics are presented as mean ± SD. An alpha level of < 0.05 was set a priori. To examine the effects of training modality and time on the outcome measures, a series of two‐way mixed analysis of variance (ANOVA) with a 2 × 3 factorial design was utilized, with group (high‐volume eccentric vs. combined training group) as the between‐subjects factor and time (pre‐training, post‐training, and detrained) as the within‐subjects factor. Mauchly's test of sphericity was conducted for all within‐subjects effects, and where sphericity was violated, degrees of freedom were corrected using the Greenhouse‐Geisser epsilon. The Group × Time interaction was examined to determine whether the two training modalities produced differential adaptations across the testing periods, including the retention of training‐induced adaptations following the 2‐week detraining phase.

Where significant main effects or interactions were detected, follow‐up comparisons were conducted using paired‐sample *t*‐tests to examine within‐group changes across time points (pre‐to post‐training, post‐training to detrained, and pre‐training to detrained), and independent‐sample *t*‐tests to assess between‐group differences at each individual time point. To control for inflation of Type I errors associated with multiple comparisons, a Bonferroni correction was applied to all post hoc tests. Effect sizes are reported as partial eta‐squared (ηp^2^) for ANOVA effects, interpreted as small (0.01–0.059), medium (0.06–0.14), and large (> 0.14) following Hopkins et al. (1999). Hedges' *g* is reported for all pairwise comparisons and interpreted in line with previous recommendations, defined as: < 0.35, 0.35–0.80, 0.80–1.50, > 1.5 as trivial, small, moderate, and large, respectively (Rhea 2004).

To formally assess the comparability between protocols, equivalence between groups was additionally assessed using two one‐sided tests (TOST) on change scores (pre‐to‐post, pre‐to‐detraining, and post‐to‐detraining) for 20 m sprint time, NBPA, and rapid LLB force. Equivalence margins were defined a priori using the minimal detectable difference (MDD) extracted from previously published reliability data for 20 m sprint time (Shalfawi et al. 2012), NBPA (Dias et al. 2025), and rapid LLB force (Sundh et al. 2026b) using instrumentation comparable to that of the present study. Equivalence was concluded at *α* = 0.05 if the overall TOST *p*‐value fell below this threshold. Post hoc sample size requirements for adequately powered TOST (80% power, *α* = 0.05) were estimated using the observed pooled SD of change scores from the present sample, following recommendations to interpret non‐significant equivalence tests cautiously in the context of achieved versus required sample size (Mazzolari et al. 2022).

Continuous angular velocity waveforms were further analyzed using statistical parametric mapping (SPM) to examine whether training‐induced changes in angular knee extension velocity were localized to specific phases of the movement or distributed across the full repetition. A one‐way SPM‐ANOVA was conducted within each group to determine whether angular knee extension velocity differed significantly across the three time points. Where a significant main effect of time was detected, Bonferroni‐corrected pairwise sample *t*‐tests were performed to localize differences between specific comparisons. SPM independent sample *t*‐tests were additionally conducted at each time point to examine between‐group differences. Significant suprathreshold clusters are reported with their normalized time extent (%) and cluster‐level *p*‐values. Retrospective statistical power analysis was conducted using power1d (Pataky 2017) with noise amplitude (NAMP = 7.53) and noise full‐width‐at‐half‐maximum (NFWHM = 6.1) estimated directly from the observed data following the approach described by Luciano et al. (2021), with worst‐case values taken across all groups and time points to ensure a conservative sample size estimate. The minimum effect of interest was set to 15.2°/s, corresponding to the mean angular velocity at the inflection point between controlled descent and failure of the NHE. With a Bonferroni‐corrected alpha of 0.0167 across three post hoc comparisons, a sample size of 12 participants per group was required to achieve 80% statistical power.

## 3 Results

For all mixed ANOVAs, degrees of freedom were 1 and 31 for the Group effect and 2 and 62 for the Time and Group × Time interaction effects. Descriptive statistics are presented in Table 2, and individual participant responses alongside pairwise Hedges' *g* effect sizes are presented in Figure 3.

**TABLE 2:** Descriptive characteristics by protocol and time point.

Protocol	Variable	Pre	Post	Post‐detraining
Combined	20 m Sprint (s)	3.45 ± 0.31	3.31 ± 0.26**	3.28 ± 0.27**
	NBPA (°)	41.37 ± 14.79	36.38 ± 12.40	37.55 ± 13.36
	Rapid SL‐LLB (N)	269.75 ± 66.76	305.06 ± 79.86	298.59 ± 79.02
Eccentric	20 m Sprint (s)	3.43 ± 0.28	3.31 ± 0.27*	3.30 ± 0.30*
	NBPA (°)	42.90 ± 11.55	38.86 ± 10.42	38.49 ± 10.16
	Rapid SL‐LLB (N)	274.97 ± 66.49	295.38 ± 72.92	294.35 ± 72.24

*Note:* Values are mean ± SD. NBPA = Nordic Breakpoint Angle; Rapid SL‐LLB = Rapid force produced during the isometric single‐leg long‐lever bridge (150 ms). Best value per session used: 20m Sprint and NBPA = minimum; Rapid LLB = maximum.

^*^ Significantly different from Pre (*p* < 0.05).

^**^ Significantly different from Pre (*p* < 0.001).

**FIGURE 3:**
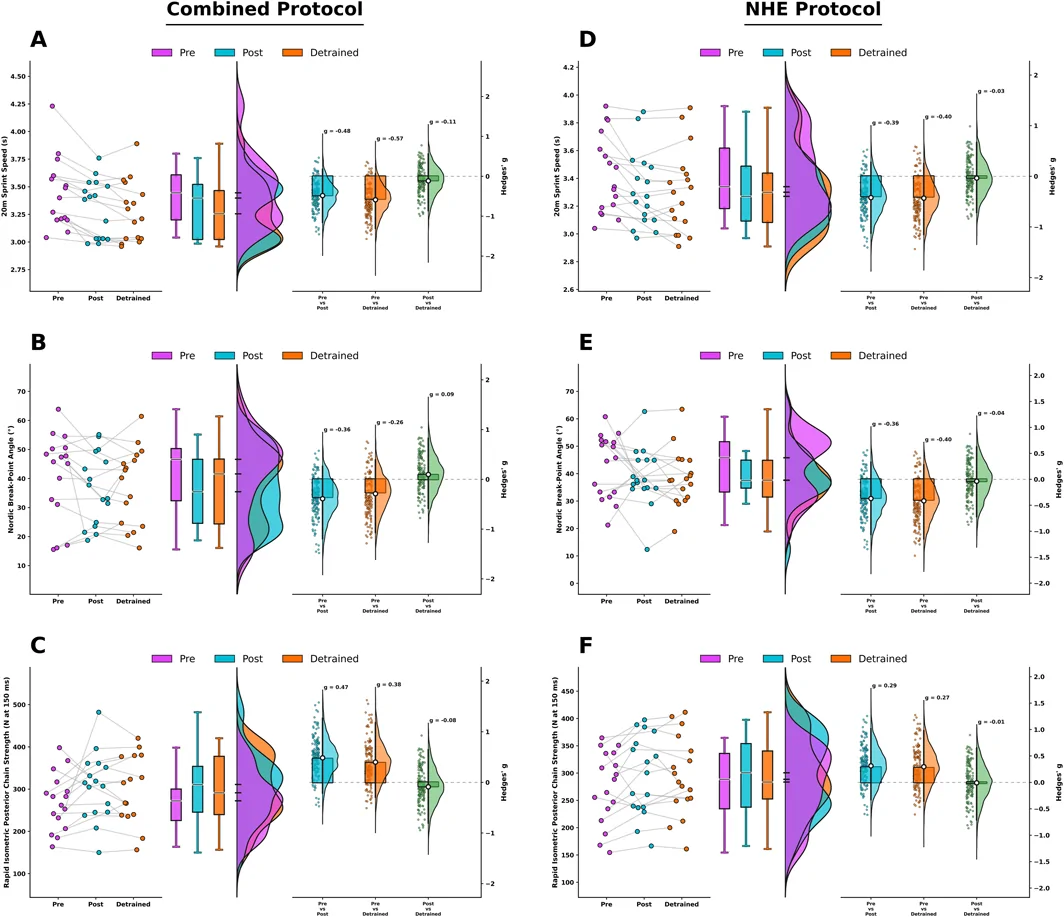
Individual and group‐level responses across time points for 20‐m sprint speed (A, D), Nordic breakpoint angle (B, E), and rapid long‐lever bridge force (C, F) for the combined protocol (A–C) and eccentric protocol (D–F). Each panel displays paired individual data points connected by lines (left), a box‐and‐whisker plot (center‐left), and a half‐violin distribution plot (center‐right). The right panel of each figure shows paired Hedges' *g* effect sizes with 95% confidence intervals derived from bootstrapped coupled estimation (5000 resamples) for Pre versus Post, Pre versus Detrained, and Post versus Detrained comparisons.

### 3.1 20 m Sprint

Both groups improved sprint performance across the testing period, with a significant main effect of Time (*F* = 33.56, *p* < 0.001, ηp^2^ = 0.520) but no significant main effects of Group (*F* = 0.001, *p* = 0.973, ηp^2^ = 0.000) or Group × Time interaction (*F* = 0.865, *p* = 0.426, ηp^2^ = 0.027). Both groups demonstrated significant but small improvements from pre‐to post‐training and pre‐to post‐detraining, with adaptations retained following the detraining period and no significant changes observed from the post‐training to the detraining period. No significant differences between the groups were observed at any time point. Equivalence testing supported comparable adaptations between protocols at two of the three comparison windows (pre‐post: *p* = 0.017; post‐detraining: *p* = 0.002), both of which were adequately powered; equivalence could not be statistically confirmed across the pre‐detraining window (*p* = 0.055).

### 3.2 Nordic Hamstring Break Point Angle

Both groups demonstrated improved eccentric strength through the NHE via the NBPA across the testing period, with a significant main effect of Time (*F* = 4.268, *p* = 0.018, ηp^2^ = 0.121; Greenhouse‐Geisser corrected *ε* = 0.729) but no significant main effects of Group (*F* = 0.194, *p* = 0.663, ηp^2^ = 0.006) or Group × Time interaction (*F* = 0.104, *p* = 0.901, ηp^2^ = 0.003). Despite the significant main effect of time, Bonferroni‐corrected pairwise comparisons did not reveal any significant within‐group differences across time points for either the NHE or combined group (all *p* > 0.05). No significant between‐group differences were observed at any time point. Nonetheless, effect sizes suggested that the combined group achieved small improvements following training that diminished to a trivial net gain following detraining, whereas the NHE group demonstrated small improvements that were largely maintained post‐detraining, retaining the small overall effect. Equivalence testing did not support statistical equivalence between protocols at any comparison window (pre‐post: *p* = 0.247; pre‐detraining: *p* = 0.228; post‐detraining: *p* = 0.171), with required sample sizes substantially exceeding the achieved size during the study (> 50 per group), indicating the study was not adequately powered to establish equivalence for this outcome.

### 3.3 Isometric Long‐Lever Bridge

Both groups improved rapid force production during the isometric long‐lever bridge across the testing period, with a significant main effect of Time (*F* = 6.81, *p* = 0.002, ηp^2^ = 0.180; Greenhouse‐Geisser corrected *ε* = 0.657) but no significant main effects of Group (*F* = 0.015, *p* = 0.903, ηp^2^ = 0.000) or Group × Time interaction (*F* = 0.429, *p* = 0.653, ηp^2^ = 0.014). No individual within‐group comparisons reached significance following Bonferroni correction, though small effects were observed across time points for both groups (*g* = 0.27–0.47), with no significant changes observed from post‐training to detrained. No significant between‐group differences were observed at any time point. Inspection of effect sizes indicated that the combined training group achieved small improvements following training that were largely maintained post‐detraining, whereas the NHE group demonstrated only a trivial overall improvement in rapid force production across the testing period. Equivalence testing similarly did not support statistical equivalence for rapid SL‐LLB force at any comparison window (pre‐post: *p* = 0.428; pre‐detraining: *p* = 0.303; post‐detraining: *p* = 0.081), with required sample sizes exceeding the achieved sample size (> 35 per group), again indicating inadequate power to establish equivalence for this outcome.

### 3.4 Angular Velocity Waveform Analysis

The SPM one‐way ANOVA revealed a significant time effect in angular knee extension velocity within the NHE group, with a large suprathreshold cluster spanning 65.4%–92.2% of the normalized movement duration (*p* < 0.001; Figure 4). Bonferroni‐corrected pairwise follow‐up analyses indicated that this was mainly driven by adaptations between baseline and post‐training (70.5%–85.7%, *p* < 0.001), reflecting a greater eccentric capacity through the terminal phase of the movement, where hamstring demand is the greatest (Figure 5). No significant differences were detected between baseline and detrained or post‐ and detrained subjects, suggesting that training‐induced adaptations were not retained following the 2‐week detraining period.

**FIGURE 4:**
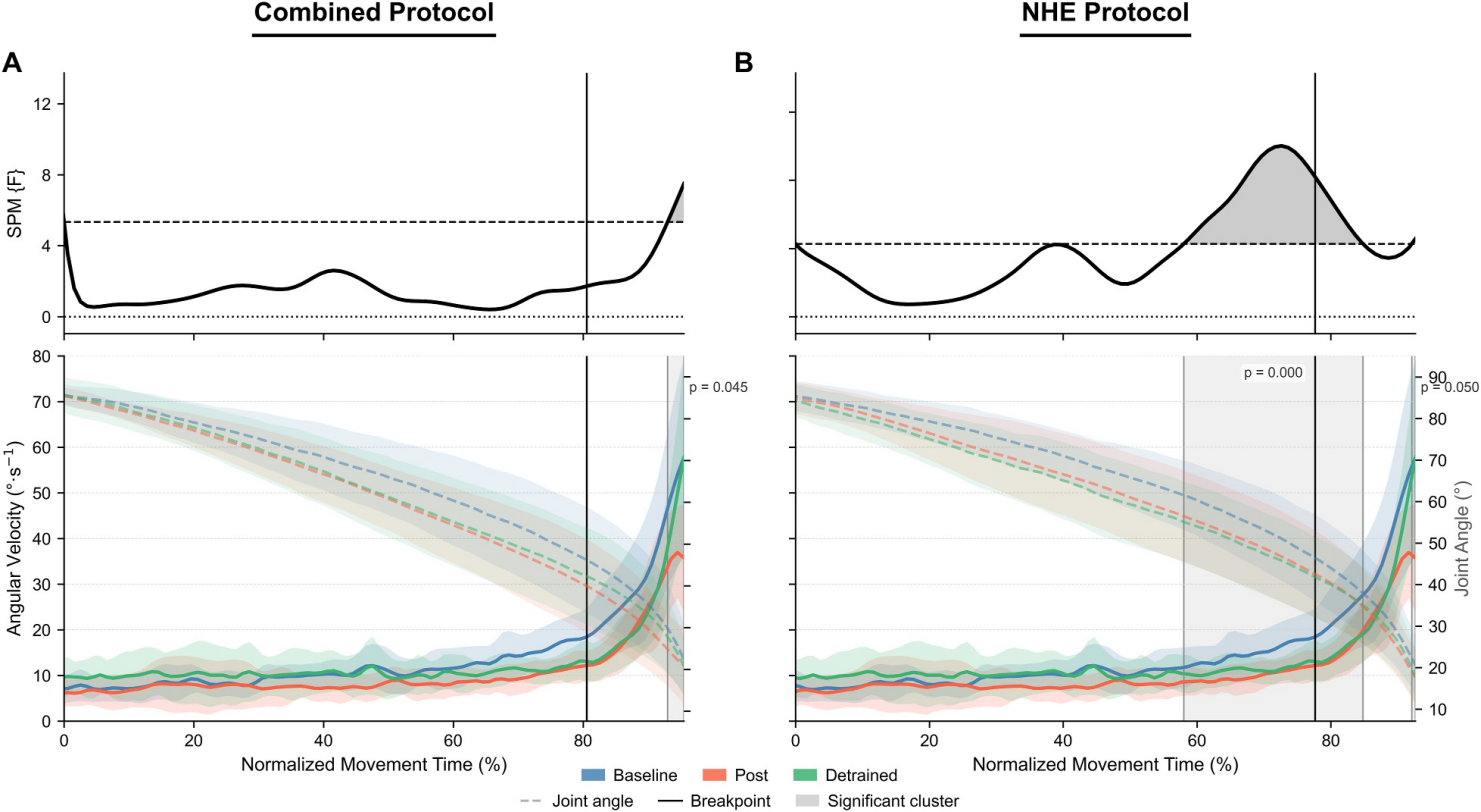
Statistical parametric mapping (SPM) of angular knee extension velocity during the NHE across training time points. SPM statistics (upper) and corresponding mean ± SD angular knee extension velocity waveforms (lower) for the combined training group (A) and the NHE group (B) across baseline (blue), post‐training (orange), and detrained (green) time points. Dashed lines represent mean joint angles. The vertical solid line denotes the breakpoint angle. Shaded regions indicate suprathreshold clusters of significant time‐effects. Dashed horizontal lines in the SPM panels represent the critical threshold determined by the random field theory.

**FIGURE 5:**
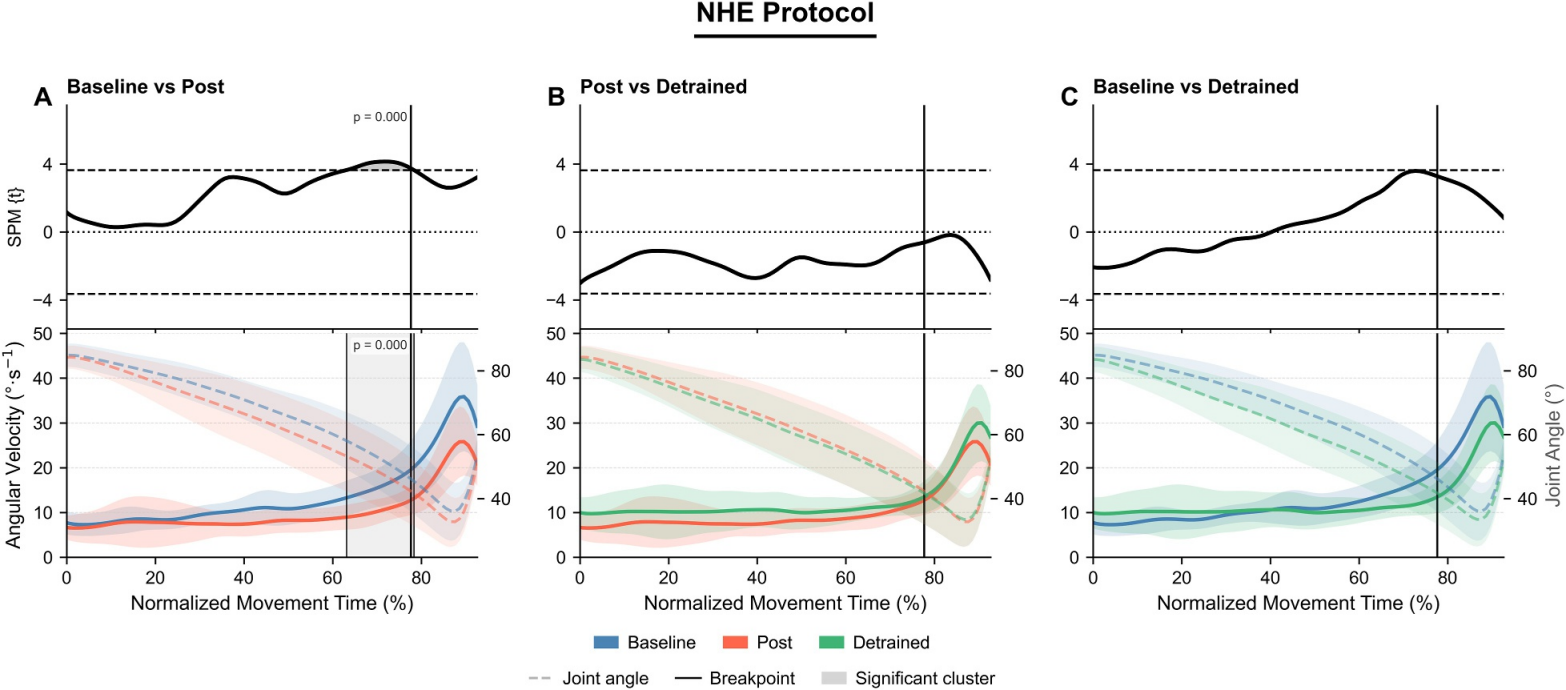
Bonferroni‐corrected pairwise SPM *t*‐tests of angular knee extension velocity during the NHE within the eccentric training group. SPM statistics (upper panels) and corresponding mean ± SD angular knee extension waveforms (lower panels) for pairwise comparisons within the NHE training group, baseline versus post‐training (A), post‐training versus detrained (B), and baseline versus detrained (C). Dashed lines represent mean joint angles. The vertical solid line denotes the break point angle. Shaded regions indicate suprathreshold clusters following Bonferroni correction (α = 0.017). Dashed horizontal lines in the SPM panels represent the critical threshold determined by the random field theory at the Bonferroni‐corrected alpha level.

Within the combined group, the SPM ANOVA did not identify any significant time effects across the primary movement. A small suprathreshold cluster was detected at 97.5%–100%, *p* = 0.045, with Bonferroni‐corrected follow‐up analyses localizing this to differences between Baseline and Post‐training (98.2%–100%, *p* = 0.016). However, this cluster is confined to the terminal boundary of the normalized movement and corresponds to the period following muscular failure. As such, this finding is not interpreted as a meaningful training adaptation. No significant between‐group differences in angular knee extension velocity were detected at any time point across the normalized movement duration.

## 4 Discussion

The primary aim of this study was to compare the effects of a higher‐volume eccentric training protocol and a combined low volume eccentric plus isometric training protocol on sprint speed, eccentric, and isometric hamstring strength across a 6‐week intervention and 2‐week detraining period. Overall, the results of the present study paint a relatively nuanced picture regarding assessment specificity in terms of interchangeability between protocols. Specifically, sprint performance appears to be the outcome for which the two approaches can most confidently be considered comparable, suggesting that a lower‐volume combined protocol may be a viable alternative when the primary training goal is the development of sprinting performance. The picture is less clear however for the underlying strength qualities captured by the NBPA and rapid SL‐LLB force. As indicated by the similar effect sizes, the pattern of change across groups did not point toward any divergence between training protocols; however, equivalence could not be formally established for either outcome, and the present study was not sufficiently powered to confidently rule out a meaningful difference at the pre‐specified margins. Overall, while the discrete outcomes appear, at face value, broadly similar between protocols, this apparent similarity should be interpreted with appropriate caution. Although the present study was adequately powered to detect the originally hypothesized between‐group differences, equivalence testing inherently demands substantially larger sample sizes to confidently establish that the two conditions do not differ beyond their pre‐specified margins, and the present study falls short of this requirement for the NBPA and rapid SL‐LLB force. As such, the present design cannot statistically distinguish true equivalence from an underpowered null result for these measures. Therefore, sprint performance remains an outcome for which comparability between protocols is best supported by the present findings, while conclusions regarding eccentric and isometric strength adaptations remain inconclusive. Beyond the discrete outcome measures, athletes in the NHE group demonstrated a significant reduction in angular knee extension velocity through the terminal phase of the movement following training, the precise range in which BF_LH_ loading is considered greatest (Van Hooren et al. 2022), indicative of greater eccentric control and load tolerance at the most vulnerable position. This adaptation was not evident in the combined training group, suggesting that the volume of eccentric‐specific work, rather than overall training volume per se, may be a more critical determinant of this particular neuromuscular adaptation. However, because the two protocols differed simultaneously in eccentric volume, exercise modality, and muscle action, the present design cannot isolate eccentric‐specific volume as the sole determinant; thus, it is plausible that the terminal range adaptation was caused due to greater exercise specificity. Furthermore, the observed adaptations in sprint speed, isometric hamstring strength, and Nordic break‐point angle appeared to be largely retained following a 2‐week detraining period (Figure 3), indicating that short‐term training cessation may not be sufficient to reverse these adaptations. Notably, the NHE group tended to better maintain eccentric‐specific adaptations, whereas the combined group showed greater retention of isometric performance, likely reflecting the specificity of the preceding training stimulus. Moreover, and perhaps most importantly, the capacity to resist eccentric loading through the terminal range, where hamstring demands are considered greatest, may be more sensitive to the removal of the specific eccentric training stimulus, suggesting that continued exposure to higher‐volume eccentric loading may be necessary to maintain this adaptation. This may in part explain the well‐documented reductions in HSI incidence observed in teams that consistently incorporated the NHE throughout the competitive season (Cadu et al. 2022; Kate and Morgan 2017), whereby the maintenance of a chronic eccentric stimulus may preserve the capacity to tolerate high eccentric forces, thereby sustaining the protective adaptations considered central to HSI risk mitigation (Andrews, Shield, et al. 2025).

The small effects observed across both eccentric and isometric hamstring strength in both groups across the intervention period are broadly consistent with the previous literature. Carmichael et al. (2022) reported trivial‐to‐small effects for both NHE eccentric strength and isometric hip extension strength following a 6‐week training period, suggesting that the magnitude of change observed in the present study may slightly exceed those of previously reported values in eccentric strength‐based adaptations but are more comparable under isometric conditions. In contrast, Presland et al. (2018) reported large adaptations in eccentric hamstring strength following both high‐ and low‐volume NHE protocols, with the low volume condition surprisingly producing notably greater effects than the high‐volume condition. Moreover, Presland et al. (2018) reported no significant changes in eccentric hamstring strength following the detraining period in either condition, suggesting that adaptations acquired through NHE training may be relatively resistant to short‐term training cessations regardless of the volume employed, a finding relatively consistent with the present study, particularly with respect to break‐point angle and the movement‐specific neuromuscular adaptations observed in the terminal portion of the NHE. However, both the high‐ and low‐volume participants in the Presland study conducted 96 total repetitions over a 2‐week duration prior to their intervention split, which likely augmented the total training stimulus and may partially explain the substantially larger effect sizes reported. Furthermore, the much larger magnitudes of change reported by Presland et al. (2018) relative to the present study may be partially attributable to the methodological difference in the assessment of eccentric hamstring strength. Presland et al. (2018) employed a field‐based force measurement device, providing a direct and sensitive measure of hamstring force production, whereas the present study utilized a camera‐based break point angle approach, which may be inherently less sensitive to detecting change and precludes direct comparison of effect size magnitudes across studies. Further contextualizing these findings, Timmins et al. (2024) reported a large increase in eccentric strength following a 38‐week intervention across both eccentric and isometric training groups, a substantially longer duration than the 6‐week duration employed in the present study. Intriguingly, however, in contrast to the present study, both groups within the Timmins et al. (2024) intervention finished the intervention significantly weaker isometrically than at baseline, a finding that likely reflects the considerable demands of concurrent training with match play across a prolonged season, which were absent in the present study. Nonetheless, the observation of meaningful strength adaptations across both modalities within only 6 weeks for the present study is encouraging, suggesting that relatively short‐term exposure to either eccentric or combined eccentric and isometric training may be sufficient to elicit practically relevant adaptations, even in the absence of extended training durations.

Beyond the strength‐based adaptations observed across the intervention period, perhaps the most practically meaningful finding of the present study is the significant improvement in 20‐m sprint performance across both training interventions, given that sprint speed represents a direct and ecologically valid measure of athletic performance and a definitive key performance indicator, with sprinting identified as a frequent action preceding goal‐scoring situations in soccer (Faude et al. 2012; Martínez‐Hernández et al. 2023, 2024). Notably, both groups demonstrated significant improvements following the intervention period and largely maintained these adaptations across the detraining phase. These findings are particularly noteworthy given the absence of a dedicated sprint program within the present study and contrast with the findings of Mendiguchia et al. (2020), who reported an unfavorable increase in 20‐m sprint times following 6‐weeks of NHE training when complementing regular soccer practice. More consistent with the sprint improvements observed in the present study, however, Ripley et al. (2023) reported moderate improvements in 20‐m sprint performance across both NHE and sprint training groups following a 7‐week intervention, with effect sizes more closely comparable to those observed in the combined protocol following detraining in the present study. Collectively, these findings suggest that posterior‐chain‐focused training, whether eccentric, isometric, or combined, likely holds meaningful potential to transfer to sprint performance, with greater adaptations likely contingent on the duration and volume of exposure.

In applied sporting environments, short‐term disruptions to training are commonplace, making the retention of training‐induced adaptations during periods of cessation a practically important consideration. Encouragingly, across all discrete outcome measures, post‐training to detraining effect sizes were at worst trivial for both groups, with eccentric hamstring strength in particular appearing to continue adapting to a small extent during the detraining periods, suggesting that the neuromuscular adaptations accrued across the 6‐week intervention were not only retained but may have continued to develop beyond the cessation of the training stimulus. A potential explanation for this continued adaptation may lie in the relatively high training volume employed across the intervention period, whereby the accumulating fatigue may have partially masked the true extent of the adaptation at the post‐intervention time point. Following the cessation of training, the dissipation of this residual fatigue may have allowed underlying compensatory adaptation to become apparent, consistent with the principles of the general adaptation syndrome (Cunanan et al. 2018; Selye 1950). This is particularly encouraging in the context of the existing literature, which generally suggests that even brief periods of training cessation may result in meaningful reductions in hamstring strength (Andrews, Pai, et al. 2025; Yamashita et al. 2023), and architecture (Carmichael et al. 2022; Pollard et al. 2019; Presland et al. 2018). Specifically, Andrews, Pai, et al. (2025) reported significant but trivial reductions in eccentric strength following a 3‐week detraining period, whereas Yamashita et al. (2023) reported a significant large reduction in NHE knee flexion torque following just 2 weeks of training cessation. Notably, however, these findings are not entirely inconsistent with the present study, as whilst global discrete outcomes appeared largely robust to detraining, the capacity to maintain eccentric loading through the terminal range did appear to revert following training cessation, a particularly important observation with potential implications toward HSI risk, suggesting that more specific neuromuscular qualities may remain sensitive to short‐term detraining even when broader strength outcomes are preserved. This dissociation between global and range‐specific adaptations highlights the potential inadequacy of discrete outcome measures alone in capturing the full response to both training and detraining and likely underscores the importance of maintaining a moderately high chronic eccentric stimulus to preserve protective qualities most relevant to HSI risk mitigation (Andrews, Shield, et al. 2025). This interpretation, however, may not fully align with the repeated bout effect, whereby even limited eccentric exposure can confer protection against subsequent muscle damage lasting from weeks to months without requiring a continuous high‐volume stimulus (Nosaka 2026); this suggests that the observed reversion may reflect a loss of movement‐specific control rather than broader protective adaptation, a distinction warranting further investigation. Nonetheless, the observation that meaningful adaptations were largely retained, and in some cases continued to develop following 2 weeks of training cessation suggests that both protocols may offer a degree of durability that is practically relevant in applied sporting contexts.

The findings of the present study have several practical implications for strength and conditioning practitioners working with athletic populations. The observation that a combined low‐volume eccentric plus isometric protocol produced sprint performance adaptations comparable to a high‐volume eccentric approach, when training load is roughly equated, suggests that practitioners may not need to rely exclusively on high‐volume eccentric training to elicit meaningful sprint performance adaptations, a notion that is previously supported (Cuthbert et al. 2020). While the present data are less conclusive regarding whether this extends to underlying strength qualities, the absence of any indication of a meaningful divergent response between protocols is at a minimum encouraging for practitioners seeking flexible programming alternatives within time‐ or fatigue‐constrained environments. On the other hand, from a logistical standpoint, both exercises present distinct practical considerations in large team‐based settings. The NHE requires relatively minimal equipment and can be set up rapidly across large squads, although it is typically associated with considerable discomfort and perceived fatigue amongst participants (Bahr et al. 2015; Ekstrand et al. 2023). Conversely, whilst the SL‐LLB may be generally well tolerated in comparison, the requirement for adequately loaded anchoring points across multiple stations may present logistical challenges in larger squad environments, and may introduce a time‐consuming element to session preparation (Sundh et al. 2026). Furthermore, whilst the importance of maintaining a chronic eccentric stimulus for HSI risk mitigation as described above should not be overlooked, it is worth noting that in contexts where sprint exposures remain relatively high, the eccentric demands inherent to high‐speed running may itself provide a sufficient stimulus to preserve these protective adaptations (Mendiguchia et al. 2020). Nonetheless, the observed sprint performance improvements across both intervention groups in the absence of a dedicated sprint program suggest that posterior‐chain focused resistance training alone may offer a meaningful transfer to athletic performance outcomes, with practical relevance for practitioners seeking to develop multiple physical qualities simultaneously within limited training time.

### 4.1 Limitations

A few limitations of the present study warrant consideration. First, although the two training protocols were designed for comparative purposes, they are not theoretically equivalent in their neuromuscular demands. The combined protocol incorporated a single‐leg isometric exercise, whereas the high‐volume eccentric protocol utilized only the bilateral NHE throughout the intervention period. This distinction means that the two groups are not directly comparable, potentially influencing the observed between‐group differences, especially during the sprint adaptations observed. Moreover, considering that all posterior chain training was intentionally isolated to the two prescribed exercises, with sprint training excluded from both protocols during the intervention period, it is plausible that any differences in sprint improvements between the two groups were due to the ballistic training nature of the combined intervention group, rather than a difference due to contraction mode. Although this approach was necessary to control confounding variables, it does not reflect the realities of applied training in sporting populations; this exclusion may therefore have meaningfully influenced the sprint performance improvements observed. In addition, it should be noted that both the eccentric and combined protocols utilized a supramaximal eccentric or overcoming isometric methodology, which inherently assumes maximal voluntary effort from all participants during each repetition. Given that each repetition was not objectively verified based on their baseline testing, it introduces the possibility that individual effort levels may have introduced noise into the training results that could have been accounted for with simultaneous testing during the training regimen. Finally, related to the statistical approach of the SPM waveform analysis, the sensitivity power analysis was based on a minimum effect of interest of 15.2°/s, derived from the within‐group inflection point based on angular knee extension velocity. However, the observed mean difference in angular velocity within the suprathreshold cluster between baseline and post‐training was 5.61°/s, suggesting that the present study would be underpowered to detect effects of this magnitude at the 80% power level. Waveform findings should therefore be interpreted with caution pending replication in larger samples. Moreover, the equivalence testing conducted in the present study carries its own limitations. The equivalence margins for 20m sprint time, NBPA, and SL‐LLB force were derived from published measurement errors rather than from an established, clinically meaningful effect size of interest, representing a comparatively conservative standard against which to conclude equivalence. Additionally, the reliability estimate used to derive the NBPA margin was obtained using break‐point methodology different from the individualized approach in the present study, given the absence of published reliability data for this specific approach. In turn, the present sample size was considered underpowered to formally establish equivalence for the NBPA and rapid SL‐LLB force at the specific margins, and these findings should therefore not be interpreted as evidence of meaningful difference between protocols, but rather as inconclusive with respect to equivalence.

## 5 Conclusion

The findings of the present study suggest that a combined low‐volume eccentric and isometric training protocol may produce sprint performance adaptations comparable to a high‐volume eccentric training approach, while the evidence for underlying strength qualities remains inconclusive and warrants further investigation. From an applied standpoint, these results carry meaningful practical implications for strength and conditioning professionals who are seeking flexible programming options, particularly given the widely reported burden of muscle soreness and fatigue associated with high‐volume eccentric loading. Specifically, although the present study did not directly assess soreness or fatigue, it appears plausible that NHE volume can be reduced to a relatively low dose, thereby potentially limiting the well‐documented burden of delayed onset muscle soreness and fatigue associated with high‐volume eccentric loading, while allowing for the supplementation of lengthened state resistance training, potentially isometric, without necessarily compromising sprint performance adaptations. This approach may be particularly advantageous during in‐season training periods, where managing neuromuscular fatigue and maintaining availability are the primary concerns. However, practitioners should remain cautious about excessive reductions in eccentric training volume, as maintaining a sufficiently high eccentric stimulus may be necessary to preserve the specific neuromuscular qualities most relevant to HSI risk mitigation.

## Author Contributions

A.E.S., P.C., and N.J.R. contributed to the study conception and design. Material preparation was carried out by A.E.S., A.L., P.C., N.J.R., and D.S.V. Data collection was performed by A.E.S., and D.S.V. Data analysis was conducted by AES. The first draft of the manuscript was written by A.E.S. and all authors commented on previous versions of the manuscript. All authors have read and approved the final manuscript.

## Funding

The authors have nothing to report.

## Ethics Statement

This study was conducted in accordance with the Declaration of Helsinki and was approved by the Institutional Review Board at the University of Salford (reference No: 2025‐7488‐9326, approval date: 5 August 2025).

## Conflicts of Interest

The authors declare no conflicts of interest.

## Supporting information


**Table S1:** Within‐session reliability of primary outcome measures within the present sample.

## Data Availability

The data that support the findings of this study are available from the corresponding author upon reasonable request.
