# Supplementary material for: Examining the Impact of Supramaximal Eccentric and Long‐Lever Isometric Training Protocols on Isometric Hamstring Strength, Nordic Hamstring Performance, and Sprint Speed
Source: Eur J Sport Sci. 2026 Sep 29;26(10):e70257. doi: 10.1002/ejsc.70257 (PMC13621811; doi:10.1002/ejsc.70257)
Supplement: Supplementary file 1 [file EJSC-26-e70257-s001.pdf]

Supplementary Table S1. *Within-session reliability of primary outcome measures within the present sample.*

	Relative Reliability		Absolute Reliability	
**Outcome**	**ICC(3,1) [95% CI]**	**Rating**	**CV% [95% CI]**	**Rating**
NBPA	0.86 [0.77, 0.92]	Good	10.16% [7.91, 12.40]	Moderate
LLB Rapid force (150 ms)	0.89 [0.82, 0.94]	Good	7.68% [5.80, 9.56]	Good
20 m sprint	0.99 [0.99, 1.00]	Excellent	0.49% [0.34, 0.63]	Excellent

Note. ICC = intraclass correlation coefficient; CI = confidence interval; CV% = coefficient of variation; NBPA = Nordic Break-Point Angle; LLB = Long-Lever Bridge. Rapid force was assessed at 150 ms from onset of the isometric LLB. Reliability was calculated from repeated trials within a single testing session and reflects trial-to-trial measurement variability (within-session reliability). ICC estimates reflect a two-way mixed-effects model, single rater/measurement, absolute agreement [ICC(3,1)], with ratings based on the lower bound of the 95% CI, as suggested by Koo & Li (2016): <0.50 (poor), 0.50-0.74 (moderate), 0.75-0.89 (good), >0.90 (excellent). CV% was calculated from the sample SD and rated based on the point estimate: <5.00% (excellent), 5.00-9.99% (good), 10.00-14.99% (moderate), >15.00% (poor).
